# Legacy effects in temporally separated tadpole species are not mediated by invasive Western Mosquitofish (*Gambusia affinis*)

**DOI:** 10.1002/ece3.10034

**Published:** 2023-04-19

**Authors:** Geoffrey R. Smith

**Affiliations:** ^1^ Department of Biology Denison University Granville Ohio USA

**Keywords:** amphibians, *Anaxyrus americanus*, competition, nonnative species, predator‐mediated interactions, priority effects, *Rana catesbeiana*

## Abstract

Temporally separated species are often thought to have limited competition over a shared resource. However, early arriving species may consume a limited resource such that later‐arriving species have access to fewer resources and thus experience competitive effects, even if they are temporally separated (i.e., they experience legacy effects from the early species). The presence of a predator might affect potential legacy effects by influencing the behavior or survivorship of the early species. Using a mesocosm experiment, I examined whether the presence of nonnative Western Mosquitofish (*Gambusia affinis*) mediated legacy effects in the interaction of two temporally separated species of tadpoles, early arriving American Toads (*Anaxyrus americanus*) and late‐arriving Bullfrogs (*Rana catesbeiana*). *Anaxyrus americanus* tadpoles reduced *R. catesbeiana* tadpole growth despite all *A. americanus* tadpoles metamorphosing 8 days before the introduction of *R. catesbeiana* tadpoles into the mesocosms (i.e., legacy effects). *Gambusia affinis* had limited effects on *A. americanus* (1 day delay in metamorphosis but no effect on survivorship or size at metamorphosis) and positive effects on *R. catesbeiana* (increased growth). There were no significant interactions between the *A.* americanus tadpole density and *G. affinis* treatments. In conclusion, I found evidence of significant legacy effects of *A. americanus* tadpoles on *R. catesbeiana* tadpoles, but no evidence that *G. affinis* mediated the legacy effects.

## Graphical abstract

This manuscript reports on the results of a mesocosm experiment that determined that American Toad tadpoles had density‐dependent effects on Bullfrog tadpoles even though there was no temporal overlap between the two species (the vast majority of American Toad tadpoles had metamorphosed >2 weeks before the introduction of Bullfrog tadpoles). The experiment also showed that the Western Mosquitofish did not mediate these legacy effects.

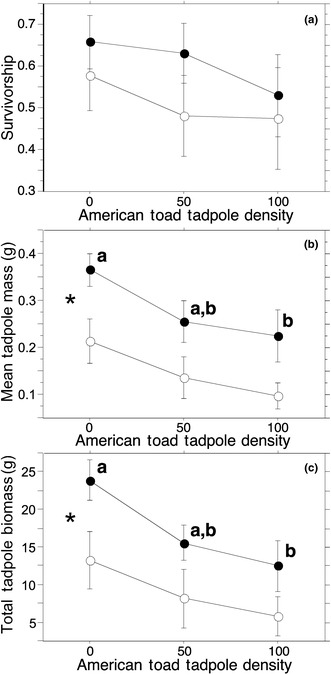

## 1 INTRODUCTION

The timing of arrival of one species relative to the arrival of a potential competitor (i.e., earlier, at the same time, or later) can determine the extent of competition between the species (e.g., Blackford et al., 2020). Temporally separated species or those that have minimal seasonal overlap may have reduced or eliminated competition over a shared resource (Lawler & Morin, 1993; Wilbur, 1980), as has been shown in a variety of taxa (McKane et al., 1990; Monceau et al., 2015; Sladecek et al., 2017). However, some temporally separated species have been shown to compete (e.g., Barnes & Murphy, 2023; Branson, 2010; Faeth, 1986; Kaplan & Denno, 2007), presumably via legacy effects which occur when the effects of a species are evident in a community even after the species is no longer present (Cuddington, 2011).

The relative timing of competitor arrival and extent of temporal overlap influences the strength, impacts, and even outcomes, of competition in anuran tadpole assemblages (e.g., Carter & Rudolf, 2019; Rudolf, 2018; Rudolf & McCrory, 2018; Rudolf & Singh, 2013). Priority effects for species with temporal overlap are relatively well studied in anuran tadpole communities (e.g., Dayton & Fitzgerald, 2005; Hernandez & Chalcraft, 2012; Knight et al., 2009; Lawler & Morin, 1993; Wilbur, 1997). Less is known about how anuran species that have no temporal overlap will compete (or not) with each other.

The breeding phenology of anurans in ponds frequently leads to tadpoles of different species occupying the same pond but not overlapping, or only partially overlapping, temporally. The presence of legacy effects of an early species affecting a later species in anuran larvae is therefore potentially important. Legacy effects have been demonstrated in larval anuran communities. For example, *Hyla andersonii* tadpoles introduced into mesocosms later in the season grew and developed slower than those introduced earlier in the season, consistent with the later season tadpoles experiencing lower resources due to consumption of resources (e.g., periphyton) by early season conspecific and *Anaxyrus woodhousii* tadpoles (Morin et al., 1990). The presence of *Anaxyrus americanus* in the spring lowered the survivorship and delayed metamorphosis of *Hyla chrysoscelis* tadpoles even though they did not overlap temporally, likely because of shifts they caused in the composition of the algal community resulting in an increase in the prevalence of lower‐quality algal species (Wilbur & Alford, 1985). *Scaphiopus holbrooki* tadpoles had a negative competitive effect on the growth of *Hyla chrysoscelis* despite no temporal overlap; however, *Anaxyrus americanus* did not (Wilbur, 1987). The presence of *Hyla crucifer* tadpoles reduced the survival and mass of metamorphosis of *H. versicolor* even though the *H. crucifer* was almost all metamorphosed before the *H. versicolor* were added (Morin, 1987). Thus, early arriving larval anuran species may be able to competitively affect later‐arriving larval anuran species through a shared resource, such as periphyton or algae, and thus reduce the growth or survivorship of the later species, even if there is no, or very limited, temporal overlap.

The importance of competitive legacy effects in communities could be mediated by predators. Previous work has demonstrated that the outcome of competition among simultaneously occurring anuran tadpole species can be altered by the presence of a predator. For example, the presence of a predator may allow competitively weaker species of tadpoles to persist in experimental communities with competitively superior species because they differentially prey on the superior competitive species or they cause a reduction in the overall density of tadpoles limiting the impact of competition (Morin, 1981, 1983; Relyea & Rosenberger, 2018; Rudolf & Roman, 2018; Smith, 2006). Second, the predator can induce a behavioral response such that the impact of the first species on a shared resource is reduced, such as through inhibition of foraging activity or through changes in morphology related to consumption (e.g., mouth parts) between the two species (Relyea, 2000; Werner, 1991; Werner & Anholt, 1996). Thus similar mediation of competitive legacy effects could arise if the early arriving species is more strongly affected by the predator than the late‐arriving species. Indeed, the presence of a predator reduced the competitive effects of an early arriving species (*Pseudacris crucifer*) on a late‐arriving species (*Hyla versicolor*) that temporally overlapped briefly as tadpoles (Morin, 1987).

One potential predator that could influence competitive legacy effects in anuran larval communities is the Western Mosquitofish (*Gambusia affinis*). *Gambusia affinis* is among the most widely introduced and successful invasive freshwater fishes (Bernery et al., 2022). Mosquitofish (*Gambusia affinis* and *G. holbrooki*) have been introduced into a variety of freshwater ecosystems around the world and can have dramatic negative effects, such as population declines or extinctions, on invaded aquatic communities, including amphibians (Pyke, 2008). *Gambusia* can also have negative effects on amphibians in their native range (Baber & Babbitt, 2003). However, the effects of fish can vary among species of amphibians based on their susceptibility to fish predation. For example, the tadpoles of the relatively unpalatable Bullfrog (*Rana catesbeiana*) can benefit from the presence of fish through the consumption of potential competitors (e.g., Werner & McPeek, 1994) or invertebrate predators (e.g., Smith et al., 1999; Werner & McPeek, 1994). Thus, the consequences of the presence of a fish predator, especially an introduced species such as *G. affinis*, on anuran larval communities can be complex.

I examined whether the presence of nonnative Western Mosquitofish (*Gambusia affinis*) mediates legacy effects in the interaction of two temporally separated species of tadpoles, early arriving American Toads (*Anaxyrus americanus*) and late‐arriving Bullfrogs (*Rana catesbeiana*). There is evidence that *A. americanus* and *R. catesbeiana* tadpoles are potential competitors over a shared algal resource (e.g., Boone et al., 2007; Seale & Beckvar, 1980). Previous studies using *A. americanus* and *R. catesbeiana* from the same source populations as those used in my experiment examined the effects of *G. affinis* on the survivorship, growth, and behavior of their tadpoles. Local *A. americanus* tadpoles did not alter activity in the presence of *G. affinis* (Smith et al., 2008, 2009). However, *G. affinis* had a negative effect on the survivorship and size at metamorphosis of *A. americanus* in a mesocosm experiment (Smith & Dibble, 2012), as did Bluegill (*Lepomis macrochirus*) (Smith et al., 2016), suggesting they are susceptible to fish predators. Local *R. catesbeiana* did not alter their activity in the presence of *G. affinis* in laboratory experiments (Smith et al., 2008, 2009). I have not previously conducted experiments on the effects of *G. affinis* on local *R. catesbeiana* survivorship; but in a mesocosm experiment, another potential fish predator, *L. macrochirus*, had no effect on the survivorship of local *R. catesbeiana* tadpoles (Smith et al., 2016). Based on the results of these previous studies, I predicted that the presence of *A. americanus* tadpoles would reduce the amount of resources (e.g., periphyton) available to the *R. catesbeiana* and thus the *R. catesbeiana* would have lower growth and survivorship in mesocosms with increasing numbers of *A. americanus* tadpoles (i.e., legacy effects). However, I also predicted survivorship of American toads would be reduced by the presence of *G. affinis* based on previous experiments on the effects of *G. affinis* on *A. americanus*. This predicted effect of *G. affinis* on *A. americanus* should therefore reduce their effect on the shared algal resource, resulting in reduced effects of *A. americanus* on *R. catesbeiana* growth and survivorship in the presence of *G. affinis* (i.e., reduced legacy effects).

## 2 MATERIALS AND METHODS

I collected parts of six *A. americanus* egg masses from Olde Minnow Pond on 20 April and parts of two *R. catesbeiana* egg masses from Wood Duck Pond and Spring Peeper Pond on 17 June. All ponds are located on the Denison University Biological Reserve, Granville, Licking Co., Ohio, USA (40°5′ N, 82°31′ W). Eggs were incubated in aged tap water at 17–19°C in the laboratory. Upon hatching, tadpoles were maintained in large plastic containers and fed ground rabbit food pellets ad libitum until introduced into the experiments as free‐swimming and feeding tadpoles (stage 25–26; Gosner, 1960).

I filled 1135 L cattle tanks (*N* = 30) with 800 L (depth = 44 cm) of well water on 19–20 April. I added deciduous leaf litter (8 L; mostly maple leaves, *Acer* spp.) to provide nutrients and structure to the mesocosm on 28 April. On 29 April, I added 3.78 L of pond water to each mesocosm to introduce zooplankton and phytoplankton, and 30 g of rabbit food pellets to provide initial resources for the tadpoles. I covered mesocosms with fiberglass window screening (1 mm^2^ mesh) to prevent colonization by macroinvertebrates and other amphibians.

The fully factorial experimental design (replicated five times) consisted of three *A. americanus* tadpole density treatments (0, 50, or 100) and two *G. affinis* treatments (0 or 6). I introduced the appropriate numbers of tadpoles of *A. americanus* (mean mass = 0.013 ± 0.008 g) to mesocosms on 1 May. I added 3 male and 3 female *G. affinis* that had been collected by dipnet from a local pond (Olde Minnow Pond) to each *G. affinis* treatment mesocosm on 3 May. The *G. affinis* were free‐swimming in the mesocosms. The mean standard length of 20 randomly selected *G. affinis* collected at the same time as the *G. affinis* used in the experiment was 19.25 ± 0.04 mm (range = 15–22 mm). To minimize stress on the *G. affinis* prior to the experiment, I did not measure the fish used in the experiment; however, I took care to minimize variation in size among replicates and treatments. I added 100 tadpoles of *R. catesbeiana* (mean mass = 0.040 ± 0.002 g) to each mesocosm on 27 June, after all American Toads had metamorphosed. Tadpole densities were at the low end of naturally occurring densities of tadpoles of these and other species observed in local ponds (e.g., Smith et al., 2003, 2005). The density of *G. affinis* used was also within the range of densities of *G. affinis* found in local ponds (range = 1–32 m^−2^; J. E. Rettig and G. R. Smith, unpublished data). In addition, I have used similar numbers of *G*. affinis in other mesocosm experiments that had high survivorship of *G. affinis* and had impacts on tadpoles, including *A. americanus* (e.g., Smith et al., 2013; Smith & Dibble, 2012; Smith & Harmon, 2019). Water levels in mesocosms were allowed to fluctuate with evaporation and precipitation.

For *A. americanus*, I allowed metamorphosis to occur, removing metamorphs daily when both forelimbs had emerged. To describe the time to metamorphosis, I used the median days to metamorphosis for each mesocosm rather than the mean because the median is less affected by any outlier individuals that would have an outsized impact on the mean. Metamorphs were housed in the laboratory in plastic containers with access to water until the tail was resorbed. I then measured their SVL to the nearest 0.01 cm with digital calipers and weighed them to the nearest 0.001 g using an electronic balance. In central Ohio, tadpoles of *R. catesbeiana* overwinter, and therefore, no *R. catesbeiana* metamorphosed during the experiment. At the end of the experiment (18 July), I removed all surviving *R. catesbeiana* tadpoles from mesocosms and weighed them to the nearest 0.001 g using an electronic balance (after blotting dry with a paper towel). I also removed *G. affinis* from the mesocosms and counted all individuals. Reproduction of *G. affinis* occurred in all mesocosms in which they had been stocked; however, the number of *G. affinis* at the end of the experiment did not differ among the *A. americanus* treatments (*F*_2,12_ = 2.05, *p* = .17).

I collected water from each mesocosm on 5 dates throughout the experiment (25 May, 3 June, 10 June, 20 June, and 11 July) and estimated total chlorophyll *a* (chl *a*) levels using standard fluorometric methods (Welschmeyer, 1994). Periphyton levels at the end of the experiment were estimated by removing periphyton from a predetermined area of the south‐facing internal mesocosm wall (15.5 cm × 22.8 cm) and weighing after air drying. The proportion of the surface area covered by filamentous algae was estimated at the end of the experiment for each mesocosm by averaging the observations of 5 individuals, one of whom was blind to the experimental design.

I used two‐way ANOVAs to examine the effects of *A. americanus* tadpole density and *G. affinis* treatments and their interaction on *A. americanus* (proportion metamorphosing, median days to metamorphosis, mean SVL of metamorphs, mean BM of metamorphs), *R. catesbeiana* (proportion surviving, mean tadpole mass, and total biomass of tadpoles), and primary productivity‐related variables (coverage of filamentous algae, periphyton mass at the end of the experiment). I used repeated measures ANOVA to analyze the effects *of A. americanus* tadpole density and *G. affinis* treatments on chl *a* levels over the course of the experiment. I used Tukey's HSD post‐hoc tests to compare means for significant effects in the ANOVAs. I transformed proportion data using an arcsine‐square root transformation. I used JMP Pro 16.2 (SAS Institute) for all statistical analyses. Means are given ±1SE.

## 3 RESULTS

### 3.1 Anaxyrus americanus

The proportion of *A. americanus* metamorphosing was not affected by *G. affinis* (*G. affinis*: 0.73 ± 0.05, *N* = 10; no *G. affinis*: 0.83 ± 0.03, *N* = 10; *F*_1,16_ = 1.98, *p* = .18). Density of *A. americanus* tadpoles also did not affect the proportion of *A. americanus* metamorphosing (50: 0.77 ± 0.05, *N* = 10; 100: 0.79 ± 0.04, *N* = 10; *F*_1,16_ = 0.045, *p* = .84). The interaction between the *G. affinis* and *A. americanus* tadpole density treatments was not significant (*F*_1,16_ = 0.083, *p* = .78).

**FIGURE 1:**
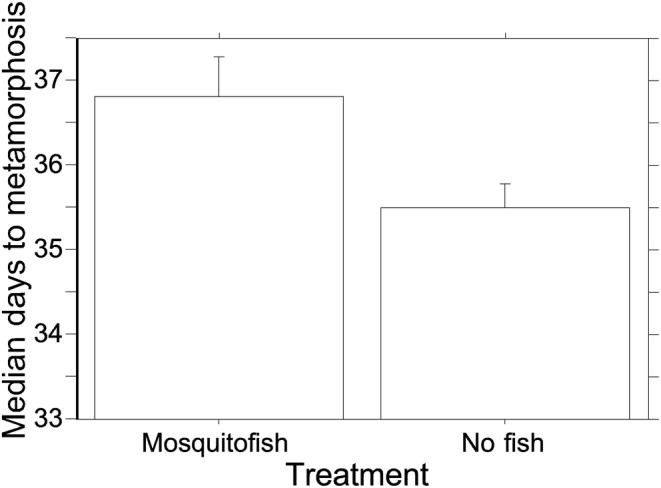
The significant effect of the presence and absence of *Gambusia affinis* on the median number of days to the metamorphosis of *Anaxyrus americanus*. Means are given ±1SE.

In mesocosms with *G. affinis*, the median number of days to metamorphosis was more than a full day later than in mesocosms without *G. affinis* (Figure 1; *G. affinis*: 36.8 ± 0.49 days, *N* = 10; no *G. affinis*: 35.5 ± 0.27 days, *N* = 10; *F*_1,16_ = 5.36, *p* = .034). Density of *A. americanus* tadpoles did not affect median days to metamorphosis (50: 36.0 ± 0.58 days, *N* = 10; 100: 36.3 ± 0.26 days, *N* = 10; *F*_1,16_ = 0.29, *p* = .60). The interaction of *G. affinis* and *A. americanus* tadpole density treatments was not significant (*F*_1,16_ = 1.56, *p* = .23).

The mean SVL of metamorphs was not affected by *G. affinis* (*G. affinis*: 1.15 ± 0.011 cm, *N* = 10; no *G. affinis*: 1.15 ± 0.013 cm, *N* = 10; *F*_1,16_ = 0.001, *p* = .98). Density of *A. americanus* tadpoles also had no effect on mean SVL of metamorphs (50: 1.14 ± 0.023 cm, *N* = 10; 100: 1.15 ± 0.016 g, *N* = 10; *F*_1,16_ = 0.11, *p* = .75). There was no significant interaction between the *G. affinis* and *A. americanus* tadpole density treatments (*F*_1,16_ = 0.011, *p* = .92).

Mean metamorph body mass was not affected by *G. affinis* (*G. affinis*: 0.21 ± 0.01 g, *N* = 10; no *G. affinis*: 0.21 ± 0.01 g, *N* = 10; *F*_1,16_ = 0.003, *p* = .96) nor by *A. americanus* tadpole density (50: 0.21 ± 0.014 g, *N* = 10; 100: 0.21 ± 0.010 g, *N* = 10; *F*_1,16_ = 0.015, *p* = .90). The interaction between the *G. affinis* and *A. americanus* tadpole density treatments was not significant (*F*_1,16_ = 0.017, *p* = .90).

### 3.2 Rana catesbeiana

**FIGURE 2:**
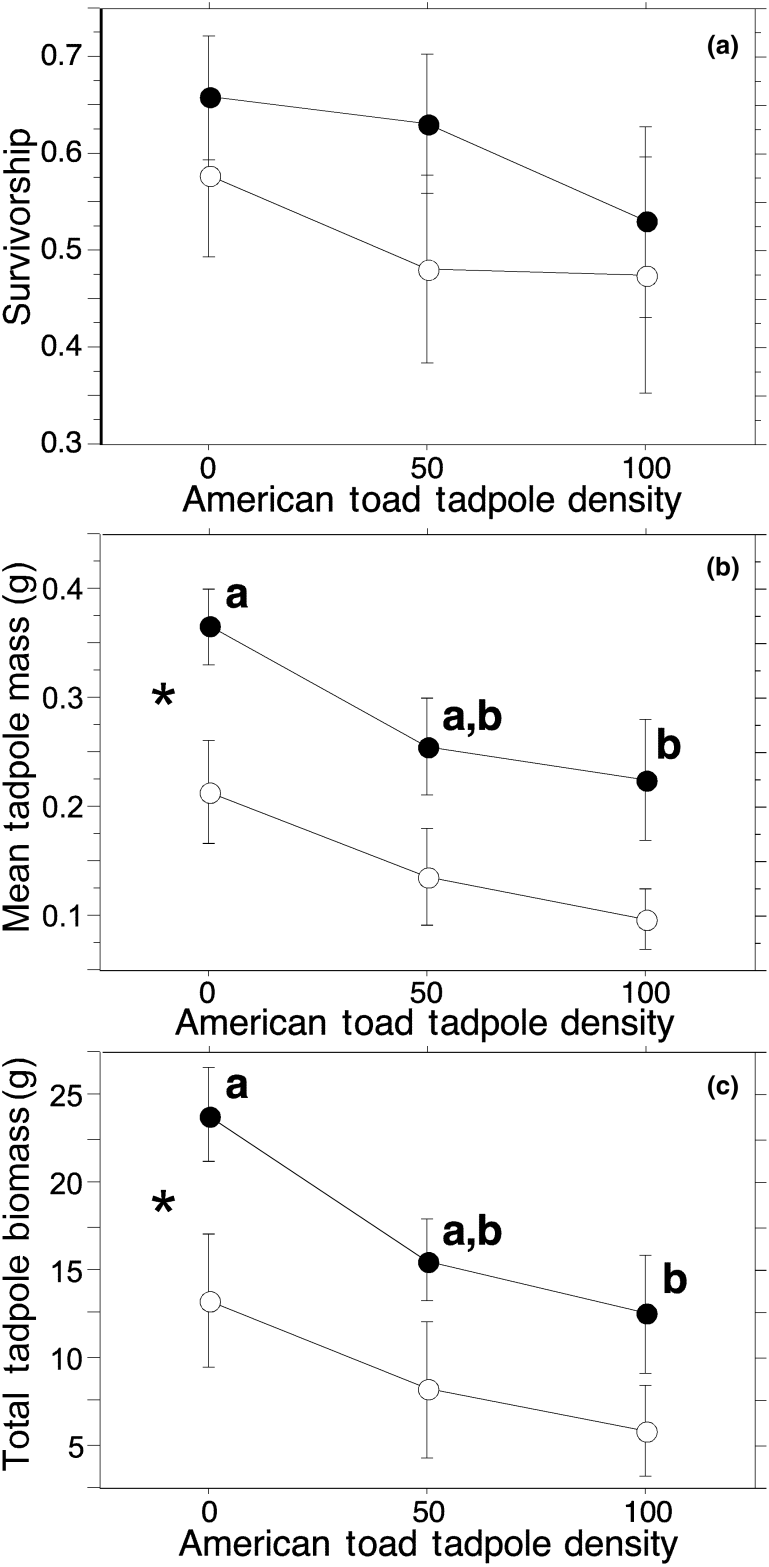
The effects of the interaction between the presence (closed circles) and absence (open circles) of *Gambusia affinis* and initial *Anaxyrus americanus* tadpole density on the (a) survivorship, (b) mean body mass, and (c) total biomass of *Rana catesbeiana* tadpoles. * Indicates a significant effect of *G. affinis* treatment. Shared letters indicate means that are not significantly different. Means are given ±1SE.

*Gambusia affinis* had no effect on *R. catesbeiana* tadpole survivorship (Figure 2a; *G. affinis*: 0.61 ± 0.045, *N* = 15; no *G. affinis*: 0.51 ± 0.056, *N* = 15; *F*_1,24_ = 1.68, *p* = .21). *Anaxyrus americanus* tadpole density had no effect on *R. catesbeiana* tadpole survivorship (Figure 2a; 0: 0.62 ± 0.052, *N* = 10; 50: 0.56 ± 0.062, *N* = 10; 100: 0.50 ± 0.075, *N* = 10; *F*_2,24_ = 0.78, *p* = .47). There was also no significant interaction between the *G. affinis* and *A. americanus* tadpole density treatments (Figure 2a; *F*_2,24_ = 0.15, *p* = .86).

*Rana catesbeiana* tadpoles in mesocosms with *G. affinis* were nearly twice as heavy on average as those from mesocosms without *G. affinis* (Figure 2b; *G. affinis*: 0.28 ± 0.029 g, *N* = 15; no *G. affinis*: 0.15 ± 0.025 g, *N* = 15; *F*_1,24_ = 14.34, *p* = .0009). As the initial density of *A. americanus* tadpoles increased, the mean mass of *R. catesbeiana* tadpoles decreased (Figure 2b; 0: 0.29 ± 0.04 g, *N* = 10; 50: 0.20 ± 0.04 g, *N* = 10; 100: 0.16 ± 0.04 g, *N* = 10; *F*_2,24_ = 4.75, *p* = .018). There was no significant interaction between the *G. affinis* and *A. americanus* tadpole density treatments (Figure 2b; *F*_2,24_ = 0.082, *p* = .92).

The total biomass of *R. catesbeiana* tadpoles produced was nearly two times higher in mesocosms with *G. affinis* compared with mesocosms without *G. affinis* (Figure 2c; *G. affinis*: 17.34 ± 1.99 g, *N* = 15; no *G. affinis*: 9.08 ± 2.03 g, *N* = 15; *F*_1,24_ = 10.18, *p* = .0039). Total biomass produced was lower in mesocosms with more *A. americanus* tadpoles at the start of the experiment (Figure 2c; 0: 18.58 ± 2.82 g, *N* = 10; 50: 11.87 ± 2.47 g, *N* = 10; 100: 9.18 ± 2.30 g, *N* = 10; *F*_2,24_ = 4.67, *p* = .019). There was no significant effect of the interaction of *G. affinis* and *A. americanus* tadpole density treatments (Figure 2c; *F*_2,24_ = 0.22, *p* = .81).

### 3.3 Primary producers

**FIGURE 3:**
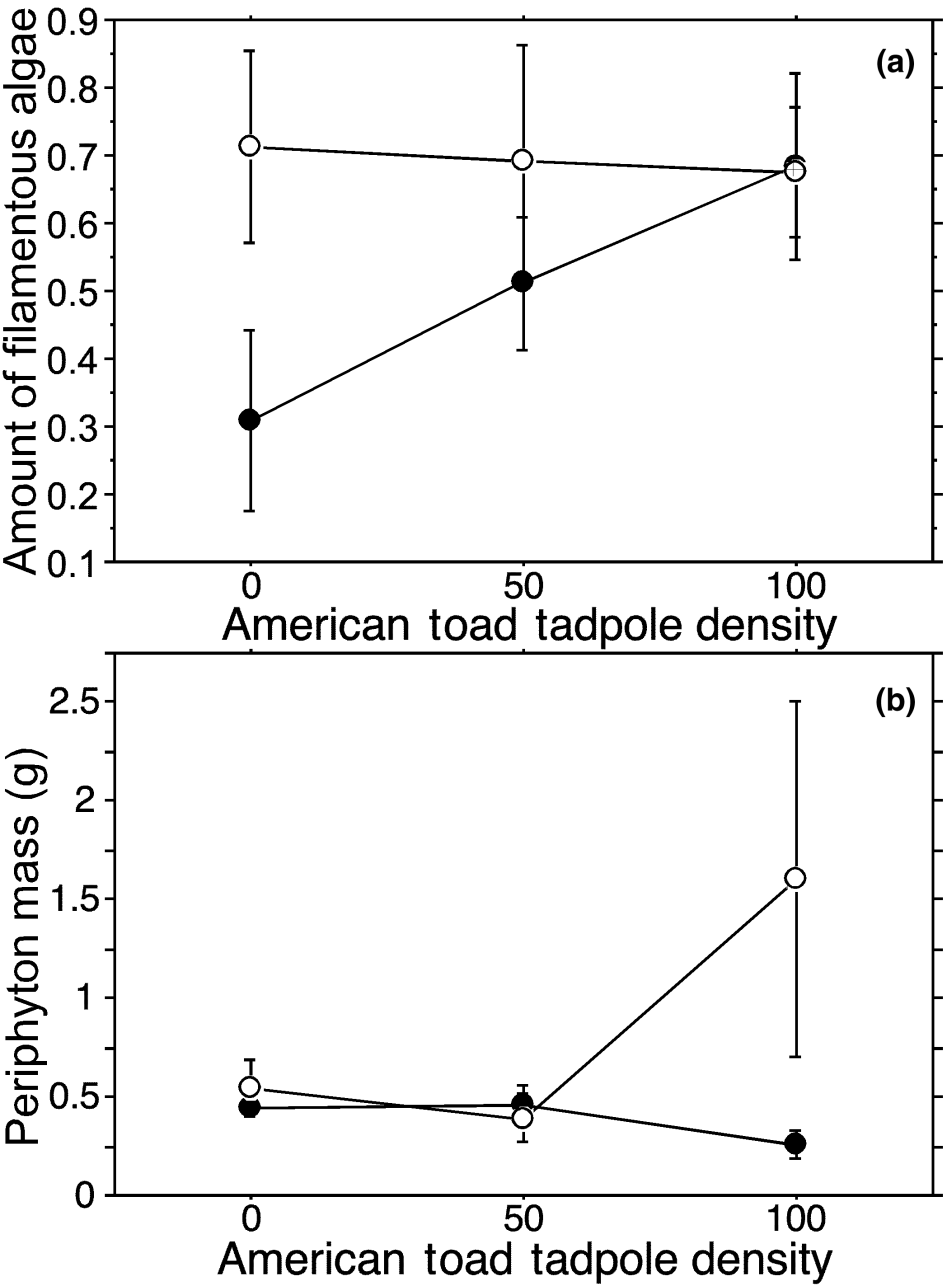
The effects of the interaction between the presence (closed circles) and absence (open circles) of *Gambusia affinis* and initial *Anaxyrus americanus* tadpole density on (a) amount of filamentous algae and (b) mean periphyton mass. Means are given ±1SE.

Mesocosms without *G. affinis* tended to have more filamentous algae than mesocosms with *G. affinis*, however, this trend was not statistically significant at the α = 0.05 level (Figure 3b; *G. affinis*: 0.50 ± 0.078, *N* = 15; no *G. affinis*: 0.69 ± 0.074, *N* = 15; *F*_1,24_ = 3.21, *p* = .086). *Anaxyrus americanus* tadpole density (0: 0.51 ± 0.11, *N* = 10; 50: 0.60 ± 0.097, *N* = 10; 100: 0.68 ± 0.079, *N* = 10; *F*_2,24_ = 0.79, *p* = .46) and the interaction of the *G. affinis* and *A. americanus* tadpole density treatments (*F*_2,24_ = 1.13, *p* = .34) had no significant effect on filamentous algae cover.

Periphyton mass at the end of the experiment was not affected by *G. affinis* (*G. affinis*: 0.38 ± 0.047 g, *N* = 15; no *G. affinis*: 0.69 ± 0.32 g, *N* = 15; *F*_1,24_ = 2.19, *p* = .15) nor by *A. americanus* tadpole density (Figure 3c; 0: 0.49 ± 0.071 g, *N* = 10; 50: 0.43 ± 0.076 g, *N* = 10; 100: 0.93 ± 0.48, *N* = 10; *F*_2,24_ = 1.03, *p* = .37). The interaction between the *G. affinis* and *A. americanus* tadpole density treatments was also not significant (Figure 3c; *F*_2,24_ = 2.07, *p* = .15).

**FIGURE 4:**
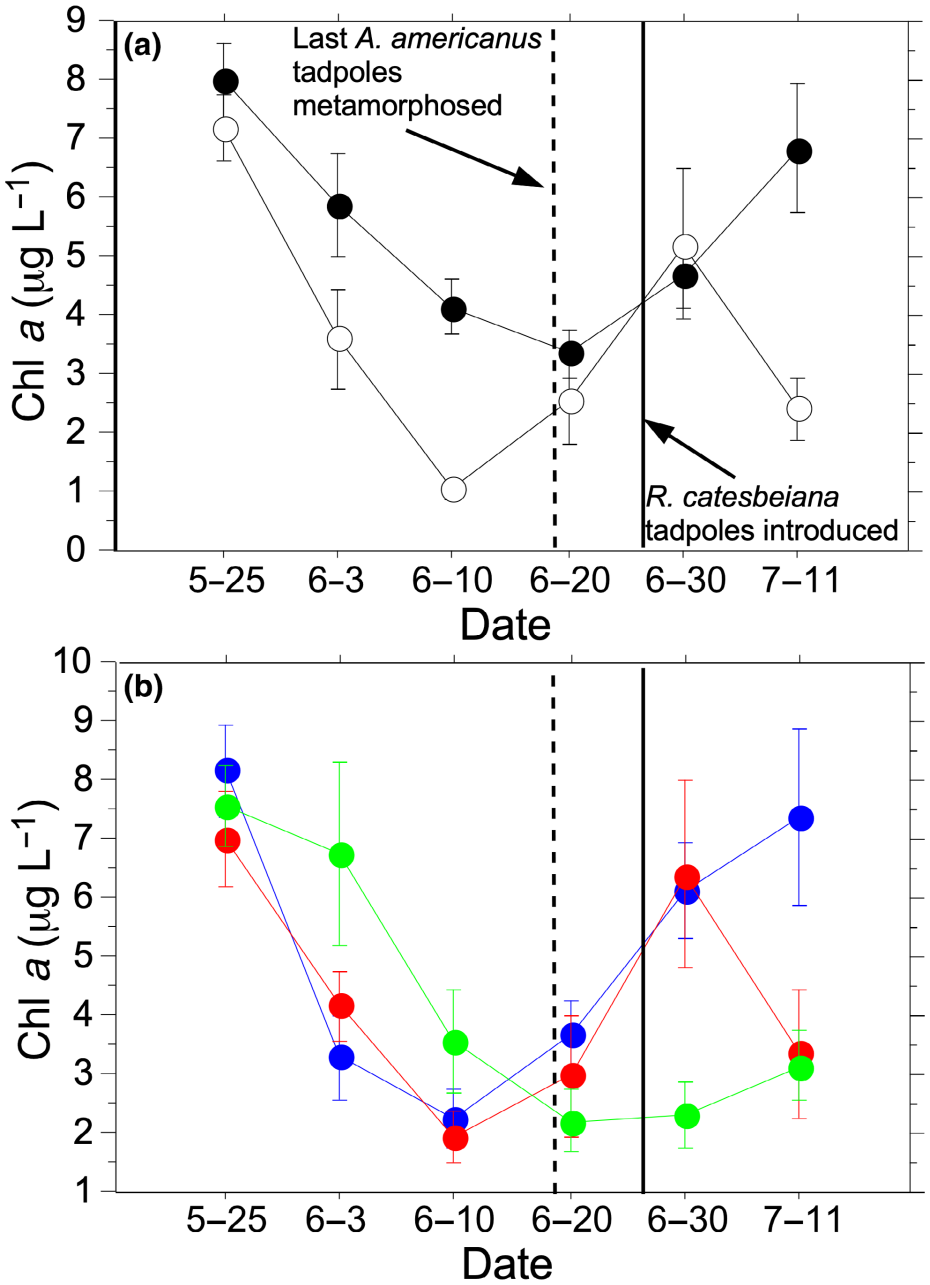
The effects of the interaction between time and (a) the presence (closed circles) and absence (open circles) of *Gambusia affinis* and (b) initial *Anaxyrus americanus* tadpole density (blue = 0 tadpoles; red = 50 tadpoles; green = 100 tadpoles) on mean chlorophyll *a* levels throughout the experiment. Dashed vertical line indicates the last day *A. americanus* collected and a solid vertical line indicates the day *Rana catesbeiana* tadpoles introduced. Means are given ±1SE.

There was less chl *a* in mesocosms with no *G. affinis* than in mesocosms with *G. affinis* (Figure 4a; *F*_1,24_ = 15.6, *p* = .0006). Overall, there was no effect of *A. americanus* tadpole density (*F*_2,24_ = 1.60, *p* = .22) or the interaction of the *G. affinis* and *A. americanus* tadpole density treatments (*F*_2,24_ = 1.45, *p* = .26) on chl *a* levels. Chlorophyll *a* levels varied over the course of the experiment (Figure 4; *F*_5,20_ = 29.9, *p* < .0001). There was a significant time * *G. affinis* treatment interaction, with the *G. affinis* treatment mesocosms had higher chl *a* levels on all dates except 20 June and 30 June when the chl *a* levels were similar between the *G. affinis* treatments (Figure 4a; *F*_5,20_ = 5.78, *p* = .002). There was also a significant time * *A. americanus* tadpole density treatment interaction with relatively similar chl *a* levels in the 0 and 50 *A. americanus* tadpole treatments but higher in 100 *A. americanus* tadpole treatments in the first third of the experiment, all treatments had similar chl *a* levels in the middle third of the experiment, and a more complex response in the last third of the experiment, with the 0 and 50 *A. americanus* tadpole treatments having higher chl *a* levels than the 100 *A. americanus* tadpole treatment on 30 June, but the 0 *A. americanus* tadpole treatment having the highest chl *a* levels on the final day of sampling (11 July) than the 50 or 100 tadpole treatments (Figure 4b; *F*_10,40_ = 4.00, *p* = .0008). The three‐way interaction between time, *G. affinis* treatment, and *A. americanus* tadpole density treatment was not significant (*F*_10,40_ = 1.87, *p* = .079).

## 4 DISCUSSION

In the simple community studied in my experiment, *A. americanus* tadpoles had a significant negative effect on *R. catesbeiana* tadpoles despite the fact that the vast majority of *A. americanus* tadpoles had metamorphosed 2–3 weeks, and all had metamorphosed by 8 days, before the introduction of *R. catesbeiana* tadpoles into the mesocosms (i.e., there were significant legacy effects). *Anaxyrus americanus* tadpoles negatively affected the mean individual mass and total biomass of *R. catesbeiana* tadpoles at the end of the experiment, but did not affect their survivorship. The negative effects of the early arriving *A. americanus* tadpoles on later‐arriving *R. catesbeiana* tadpoles that we observed are consistent with the competitive effects of *A. americanus* on later‐arriving tadpoles in other studies (Distel & Boone, 2011), including when they did not overlap (Wilbur & Alford, 1985).

It seems likely that the effect of *A. americanus* on *R. catesbeiana* arose through the consumption of potential shared resources (i.e., algae) by the *A. americanus* tadpoles. Unfortunately, I did not measure periphyton biomass during the experiment. I cannot, therefore, determine if the lack of effect of *A. americanus* tadpoles on periphyton levels at the end of the experiment is indicative of the levels experienced by *R. catesbeiana*, especially early in their time in the experiment (i.e., immediately after the *A. americanus* had metamorphosed and before the introduction of the *R. catesbeiana* tadpoles). However, other experiments have demonstrated that the consumption of algal resources by early tadpoles can lead to reduced availability of those resources to later tadpoles (e.g., Hernandez & Chalcraft, 2012; Morin et al., 1990; Rowland et al., 2017). In addition, I have found reduced periphyton levels in mesocosms with tadpoles, as well as a reduction in periphyton with higher densities of tadpoles, in other similar experiments (Smith et al., 2006, 2016; Smith & Burgett, 2012; G. R. Smith, M. Smyk, M. Jones, and J. Hollis, unpublished data). Wilbur and Alford (1985) also suggested that persistent effects of *A. americanus* on later tadpoles were likely due to an increase in the prevalence of lower‐quality algae species as a result of consumption by the *A. americanus* tadpoles. Similarly, overwintered *Rana sphenocephalus* tadpoles had a negative effect on *Bufo terrestris* tadpoles, probably due to the reduction of algal resources by the overwintered *R. sphenocephalus* tadpoles prior to the introduction of the *B. terrestris* tadpoles (Hernandez & Chalcraft, 2012). It is also possible that *A. americanus* had some other effects on the mesocosm environments in which they occurred. For example, there is some evidence that tadpoles can release growth‐inhibiting factors or otherwise condition the water in such a way that reduces the growth or survivorship of competitors (Beebee, 1995; Griffiths, 1995; Steinwascher, 1981; but see Cabrera‐Guzmán et al., 2013; Petranka, 1989, 1995).

In my experimental amphibian larval community, *G. affinis* caused a delay of slightly more than one day in the median number of days to metamorphosis in *A. americanus*, but had no effect on the proportion of tadpoles reaching metamorphosis or on size at metamorphosis. For *R. catesbeiana*, the presence of mosquitofish increased the mean mass of the tadpoles, as well as the total biomass of tadpoles produced. The presence of fish can benefit *R. catesbeiana* by the consumption of invertebrate predators (e.g., Smith et al., 1999; Werner & McPeek, 1994). *Gambusia affinis* does indeed reduce the number of invertebrate predatory species, such as odonates and dytiscid beetle larvae colonizing experimental ponds in central Ohio (Harmon & Smith, 2021). However, this effect is unlikely in our experiment since colonization of mesocosms by macroinvertebrates was prevented. Instead, I postulate that effects on primary productivity may be responsible for the positive effect of *G. affinis* on *R. catesbeiana*. The presence of *G. affinis* had significant effects on primary productivity at the end of this experiment, with more chl *a* and a tendency for less filamentous algae. Predators can indirectly positively affect prey by making nutrients more available to primary producers thus potentially increasing the resources available to the tadpoles (e.g., Costa & Vonesh, 2013). For example, *G. affinis* can affect nutrient recycling through excretion and influence primary productivity (Akhurst et al., 2012; Hargrave, 2006; Hurlbert et al., 1972). Indeed, the presence of *G. affinis* or *G. holbrooki* can increase periphyton and/or phytoplankton (Akhurst et al., 2012; Fryxell et al., 2015; Smith et al., 2013). *Gambusia affinis* can also increase primary productivity (chl *a*, periphyton) by removing primary consumers, such as zooplankton in similar mesocosm experiments (Rettig & Smith, 2021). However, the presence of *G. affinis* may not always have cascading effects on phytoplankton or periphyton (e.g., Geyer et al., 2016; Smith & Dibble, 2012). Thus, there appear to be several, not mutually exclusive, explanations of the positive effects of *G. affinis* on *R. catesbeiana* in this experiment.

Based on other studies that found negative effects of *G. affinis* on *A. americanus* (Smith & Dibble, 2012), I had expected that the presence of *G. affinis* would mediate the legacy effect of *A. americanus* tadpoles on *R. catesbeiana*. However, there were no significant interactions between the *G. affinis* and *A. americanus* tadpole density treatments on *R. catesbeiana* tadpoles. The legacy effects of *A. americanus* on *R. catesbeiana* I observed were therefore not mediated by the presence of a potential predator. The failure to find an effect of *G. affinis* on the legacy effects of *A. americanus* in my experiment is not surprising given the general lack of substantial effects of *G. affinis* on *A. americanus* survivorship, growth, and metamorphosis in my experiment. Thus, mediation of legacy effects by predators may not always be predictable from prior studies.

## AUTHOR CONTRIBUTIONS

**Geoffrey R. Smith:** Conceptualization (lead); data curation (lead); formal analysis (lead); investigation (lead); methodology (lead); project administration (lead); writing – original draft (lead).

## CONFLICT OF INTEREST STATEMENT

The author declares that he has no competing interests.

## Data Availability

The data underlying this manuscript are archived on Dryad at https://doi.org/10.5061/dryad.573n5tbcg.
